# Response to Michael Wonder’s comments on the article ‘Assessment of the therapeutic value of new medicines marketed in Australia’

**DOI:** 10.1186/2052-3211-6-8

**Published:** 2013-10-10

**Authors:** Agnes I Vitry

**Affiliations:** 1 Quality Use of Medicines and Pharmacy Research Centre, Sansom Institute for Health Sciences, School of Pharmacy and Medical Sciences, University of South Australia, Adelaide Australia

## 

We thank Michael Wonder for his comments on our article ‘Assessment of the therapeutic value of new medicines marketed in Australia’ which give us the opportunity to clarify some points further. We agree with him that the definition of pharmaceutical products which are ‘c*onsidered innovative as long as they are new and the success of innovation is defined in terms of sales, with the assumption that higher sales is a measure of the intrinsic worth of the innovation*’ is more befitting of a pharmaceutical industry perspective than a standard definition used in economics. However, the confusion between ‘*new*’ and ‘*innovative*’ is often fostered in economic reports where ‘*the conventional benchmark for measuring the pace of pharmaceutical innovation has been the total number of new molecular entities’ approved each year*’ [1]. Therefore, the main aim of our study was to demonstrate that ‘*new*’ was not equivalent to ‘*innovative*’ with regards to therapeutic value in the context of pervasive beliefs that all new medicines bring ‘*therapeutic innovation and better health outcomes*’. Wonder states that we did not present solid evidence that such beliefs are widespread in Australia. Although we have not been able to identify a recent survey on this topic in Australia, there is evidence of these beliefs in other countries. In a recent US study, 39% of participants mistakenly believed that the US FDA approves only "extremely effective" drugs; 25% mistakenly believed that the FDA approves only drugs without serious side effects [2]. There is no evidence that the Australian public is more aware than the American public that only a minority of the new medicines marketed every year provides added therapeutic value compared to existing treatments. These beliefs are strongly encouraged by the pharmaceutical industry and are relayed by public relation campaigns and the media.

Wonder comments that we are ‘*rather vague*’ on what comparisons should be made to determine the therapeutic value of a medicine. The valuation of therapeutic innovation has mainly been addressed by funding agencies such as the Pharmaceutical Benefits Scheme (PBS) in Australia that requires evidence for efficacy, safety and cost-effectiveness of a new medicine against an appropriate comptor. Medicine regulatory agencies such as the Therapeutics Goods Administration (TGA) typically require the demonstration of the efficacy of a medicine against a placebo. The two main sources of information we used in our study to inform our assessment of the therapeutic value of new medicines were the French independent medical journal Prescrire, and the Pharmaceutical Benefits Advisory Committee (PBAC)’s assessments. Both use the best standard of current care as the comptor to estimate the therapeutic value of new medicines.

Wonder comments that we seem to imply that the TGA should only register new medicines that are associated with a clear benefit and that this may lead to only one registered medicine in a given pharmacological class. This is not our point. We argued that the TGA and other regulatory agencies should only register medicines that have been shown to be equally effective and safe as previously registered medicines. The current approval system allows the registration of medicines that may be less effective or less safe than existing medicines but may be heavily promoted as ‘*newer*’ and explicitly or implicitly ‘*better*’ medicines.

Wonder states that there is ample local experience to indicate that payers do not pay more for new medicines that do not provide any additional therapeutic benefit. We acknowledge that the PBAC does its best to get good value for money for new medicines despite the strong criticisms of the industry. However, there are also some examples where the PBS paid much more for new medicines (e.g. atorvastatin) compared to other countries that choose to promote and fund the use of older, off-patent medicines such as simvastatin.

Wonder comments that we ‘*do not cite any examples where the safety of the Australian public has been compromised as a result of supposedly lax approval criteria*’. In Australia, we are fortunate to have a strong funding agency, the PBS, that imposes restrictions on use of new medicines to ensure that they are used in a safe and cost-effective manner. However, PBS restrictions may not always be implemented in practice and there are numerous examples of ‘*leakage*’ , i.e. use of medicines by health professionals outside the PBS restricted indications, often as a result of intensive marketing campaigns. For example, a large proportion of prescriptions supplied through the PBS for the ‘gliptins’ , a new class of diabetes drugs do not currently meet the criteria for PBS subsidy [3]. These medicines have not demonstrated benefits in terms of long-term health outcomes compared to older diabetes medicines and have raised serious safety concerns. The extensive use of these diabetes medicines may expose Australian patients to serious adverse effects without additional therapeutic benefit.

Finally, Wonder asks us to provide details on how stricter regulatory approval criteria would simplify the reimbursement process. Demands have be made for a strengthening of the criteria for regulatory approval including the requirement to demonstrate a minimal therapeutic improvement, in particular for cancer drugs [4,5], and requirement of active-controlled trials [6,7] that would therefore facilitate the assessment of the therapeutic value of new medicines by funding agencies [6-8].

## Competing interests

The author declares that she has no competing interest.
